# Characterization of size, shape and pattern of flow in the neo-aorta and pulmonary artery in a patient following an innovative technique of repair for truncus arteriosus

**DOI:** 10.21542/gcsp.2019.18

**Published:** 2019-09-20

**Authors:** Amr El Sawy, Mohamed Nagy, Ahmed Afifi, Hatem Hosny, Soha Romeih, Heba Aguib, Magdi Yacoub

**Affiliations:** 1 Biomedical Engineering and Innovation Laboratory, Aswan Heart Centre, Magdi Yacoub Heart Foundation, Egypt; 2 Department of Surgery, Aswan Heart Centre, Magdi Yacoub Heart Foundation, Egypt; 3 Department of Radiology, Aswan Heart Centre, Magdi Yacoub Heart Foundation, Egypt; 4 National Heart and Lung Institute, Imperial College London, UK

## Abstract

**Background.** Truncus arteriosus (TA) caries a very poor prognosis. In the absence of early correction, only 12 percent of patients born with this anomaly survive beyond one year. There is no agreement about the best method of surgical correction of this anomaly. We have devised an innovative valveless technique using autologous arterial tissue to repair TA.

**Objectives.** Characterizing the size, shape and pattern of flow in the neo-aorta and pulmonary artery, in a patient following the new technique.

**Patient and Methods.** Cardiac MRI and multislice CT imaging, followed by offline computerized image analysis was used in a patient aged 3 months, within 3 weeks of operating.

**Results.** The size, shape and topology of the neo-aorta and pulmonary artery, approximated that present in normal hearts. The pattern of flow in the reconstructed vessels was laminar, throughout the cardiac cycle with minor acceleration during systole. The pulmonary regurgitation resulting from the absence of a valve occurred during late diastole, and was well tolerated. The size of the right ventricle diminished considerably following operation, and the right ventricular ejection fraction was supernormal.

**Conclusion.** This early study in one patient provides new unique data of the size, shape, topology and pattern of flow in the neo-aorta and pulmonary artery, which appear to approximate normality. The long-term results of this promising operation need to be studied.

## Introduction

Truncus arteriosus carries an extremely poor prognosis if not corrected in infancy, with only 12% survival beyond one year^4^. Unfortunately, to date, there is no agreement about the best method of repairing this anomaly. Currently used techniques consist of separating the pulmonary artery from the truncus, followed by joining the pulmonary arteries to the right ventricle after closing the ventricular septal defect (VSD). To bridge the gap between the pulmonary arteries and the right ventricle, a variety of conduits and patches are used^1–3,5,6,8,9^. These techniques can result in severe hemodynamic disturbances both in the short and long term^7^ and require relatively frequent replacement, with its associated morbidity and mortality to consider.

To circumvent some, or all, of these problems we have developed and used an innovative technique utilizing autologous vascular tissue. This technique is designed to refashion the truncus arteriosus into neo-aorta and neo-pulmonary arteries with attempts of normalizing the pattern of flow to the aortic arch and pulmonary artery branches. In addition, it recreates a sinotubular junction which could improve truncal valve regurgitation. We describe, for the first time, the early effects of this technique on the size, shape and pattern of flow in the neo-aorta and pulmonary artery.

## Patient and Methods

A three-month-old female patient (BSA = 0.3 m ^2^) was diagnosed as truncus arteriosus type I, with an arterial oxygen saturation of 87% and a thickened truncal valve with maximum pressure gradient across 30 mmHg and moderate regurgitation. The pulmonary artery branches were of good size. Biventricular function was normal despite marked left and right ventricular hypertrophy and dilatation. The follow up imaging was done within the first three weeks after the operation.

### The new technique, in short

The new technique consists of transecting the truncus above and below the origin of the pulmonary artery (PA), followed by anastomosis of the ascending aorta to the root of the truncus. The mobilized segment of the truncus carrying the origin of the PA is used to refashion a new RV outflow which is anastomosed to a window on the anterior surface of the RV. The outlet VSD is closed through the same window to refashion a new LV outflow joining the LV to the neo-aorta.

### Image acquisition and 3D reconstruction

Imaging was performed using a 128 dual source multi-slice detector CT scanner (Siemens Somatom Definition Flash, Erlangen, Germany) using retrospective ECG triggering. Bolus tracking was done in the proximal trunk. Imaging parameters used for scanning include: detector collimation of 128 × 0.6 mm, pitch of 0.2, gantry rotation time of 280 ms, tube voltage of 80–120 kV and fully automated real-time anatomy-based dose regulation (CARE Dose 4D) to reduce radiation exposure.

The size and shape of the vessels and the topology was analyzed using Mimics Research version 21 and 3-matic (Materialise, Leuven, Belgium).

CMR was performed with 1.5 T scanner (Siemens Sonata, Erlangen, Germany) using retrospective ECG triggering. SSFP end- expiratory breath-hold cines were acquired for LVOT and RVOT view on 2 orthogonal planes, with slice thickness of eight mm. The temporal resolution was 21 ± 1 ms.

Pattern of flow was characterized by CMR using a 4D flow acquisition sequence and Medis^®^ research program for 4D Flow post-processing. Velocity was encoded in three orthogonal directions, and the images were acquired during free breathing by an imaging sequence with retrospective electrocardiogram gating (10% acceptance window, 30 reconstructed cardiac phases).

A computational fluid dynamic simulation (CFD) of the neo-pulmonary artery, along with its two bifurcations (right pulmonary artery and left pulmonary artery) was performed. The boundary conditions for the inlet and the two outlets were obtained from the phase-contrast magnetic resonance imaging (PC-MRI), from which the flow velocity data was extracted.

## Results

### Pre- and post-operative topology

**Figure 1.**
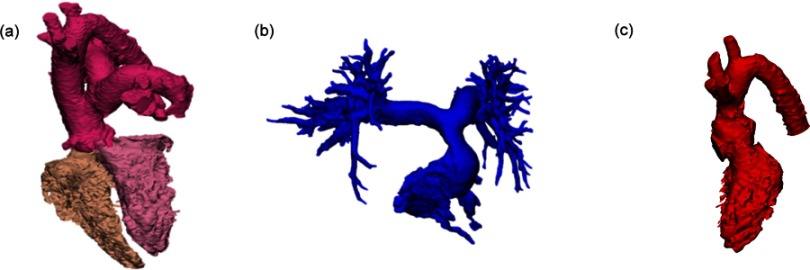
3D anatomical model showing topology of both ventricles and corresponding great arteries. Pre-operative image: position of the truncus in relation to the right and left ventricles (a), Post-operative neo-pulmonary and right ventricle (b), Post-operative neo-aorta and left ventricle (c).

This showed the position of the truncus arteriosus located on top of both the right and left ventricles and overriding the outlet VSD. The origin of the pulmonary artery was located on the left lateral aspect of the truncus immediately above the root (Figure 1a). The main pulmonary artery is directed upwards and to the left before bifurcating.

Following operation, the mobilized main pulmonary artery arises from a tailored outflow at the front of the right ventricle and is directed upwards and to the right similar to the location of a normal pulmonary artery. The preserved pulmonary artery bifurcation results in a near normal angle between the branches (Figure 1b).

Figure 1c shows the origin of the neo-aorta from the left ventricle with a short outflow tract created by the VSD patch. The ascending aorta is markedly shortened with creation of a sinotubular junction.

**Figure 2.**
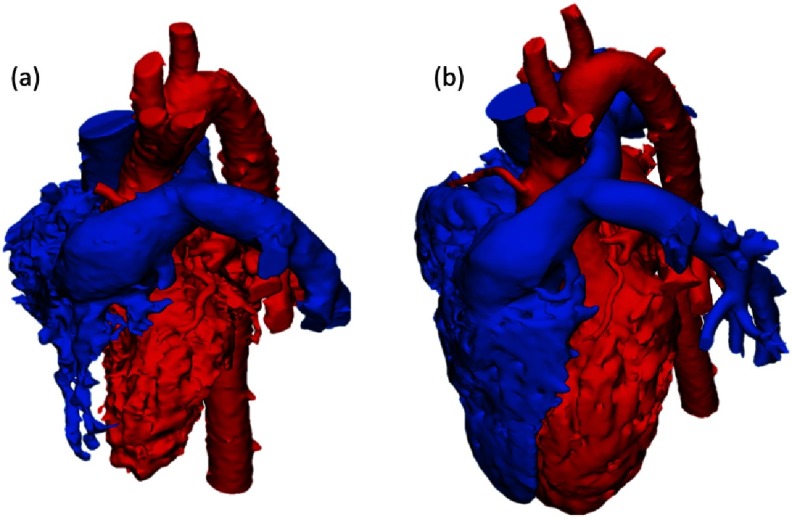
Post-operative 3D anatomical model showing the ventricular and vascular interrelation, in systole (a) and diastole (b).

The interrelation between the two ventricles and great arteries shows normal direction and crossing of the neo-aorta and pulmonary artery (Figure 2).

### Shape and size of the arteries

**Figure 3.**
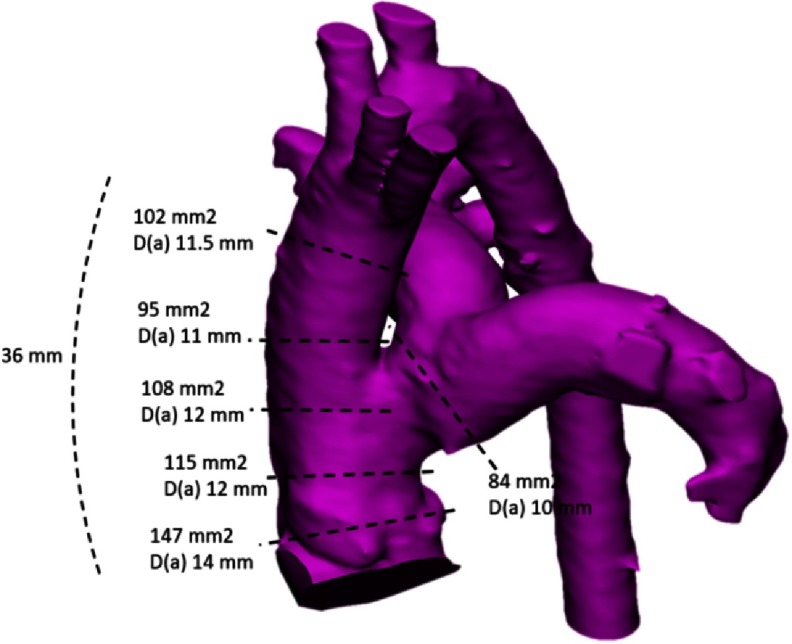
Shape and measurements of the truncus arteriosus.

Preoperatively, the truncus consisted of a bulbous root with absence of a sinotubular junction. A short main pulmonary artery arose from the common trunk 2 mm above the top of the intercoronary commissure producing marked distortion in the shape of the truncal outflow. The ascending aorta immediately distal to the pulmonary ostium is elongated (measurements, Figure 3).

**Figure 4.**
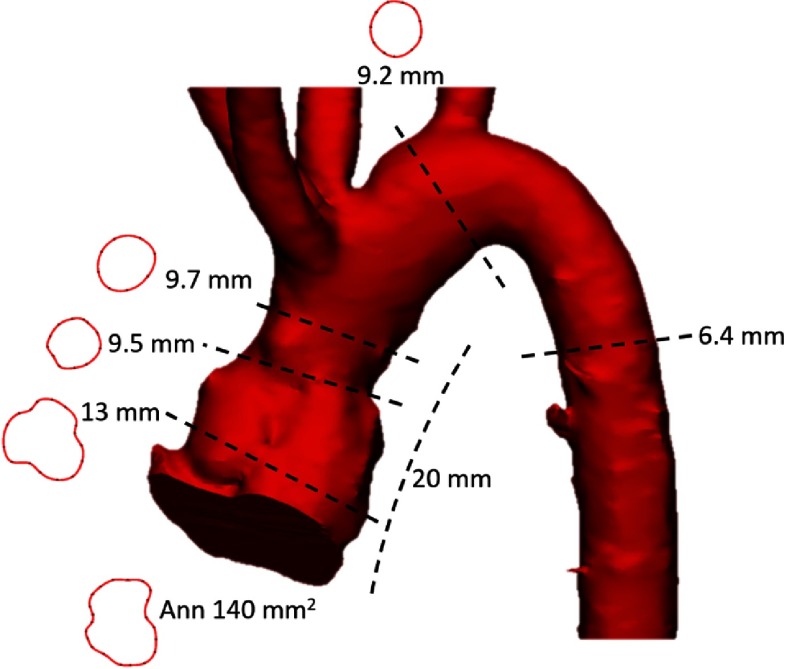
Shape and measurements of the neo-aorta.

**Figure 5.**
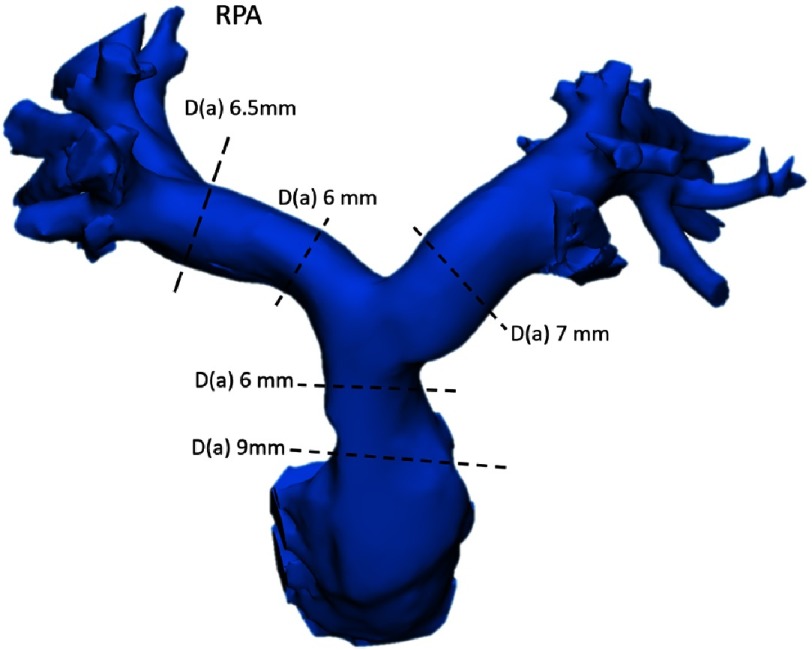
Shape and measurements of the neo-pulmonary artery, D(a) is diameter based on area measurement.

Post-operatively the neo-aorta has accentuated sinotubular junction with a markedly shortened ascending aorta (Figure 4). The neo-pulmonary artery configuration and measurements are reduced to near-normal (Figure 5).

## Post-Operative Pattern of Flow in the Arteries (4D)

### Neo-aorta

**Figure 6.**
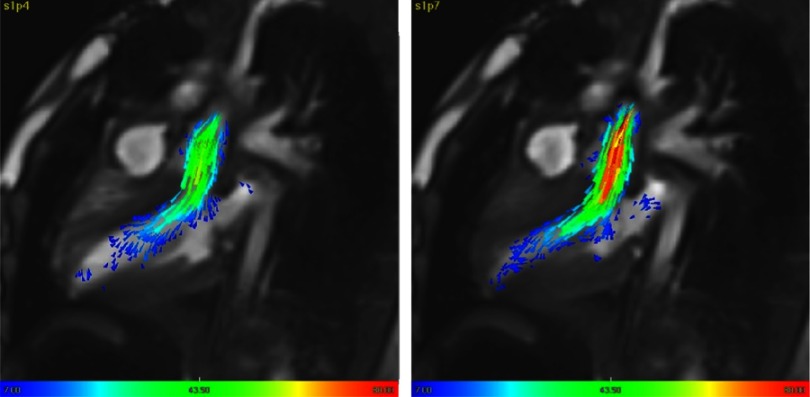
4D Pattern of flow of the neo-aorta at: early systole (left) and peak systole (right).

This showed laminar flow in early systole with slight acceleration during peak systole without evidence of disturbed flow (Figure 6).

### Neo-pulmonary artery

**Figure 7.**
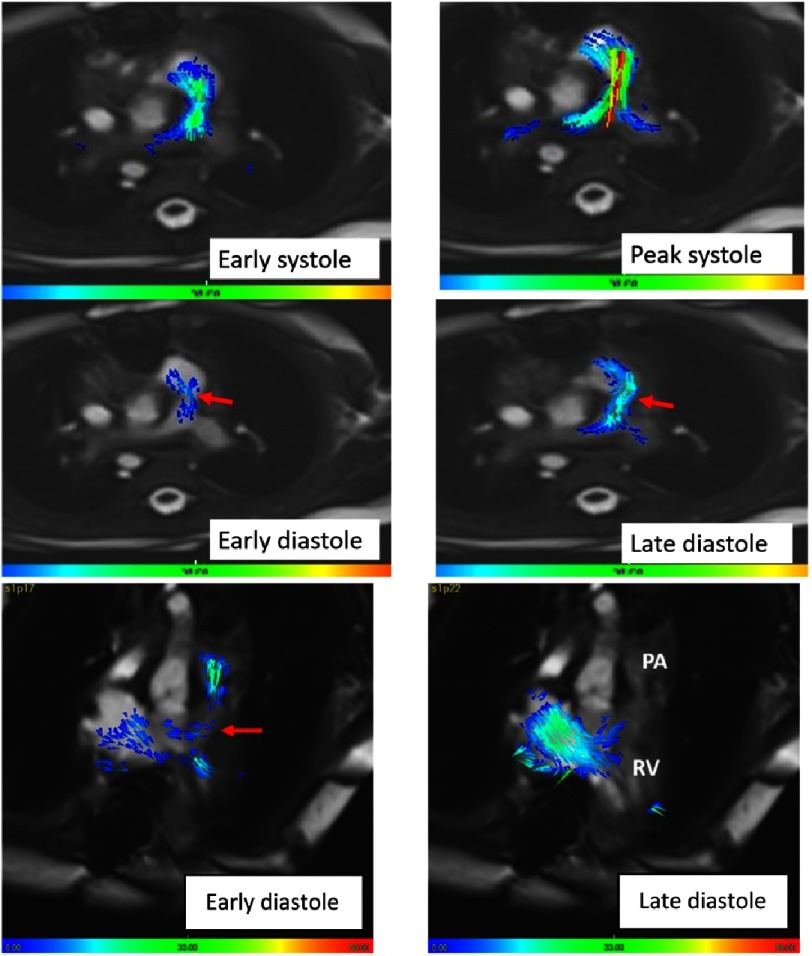
4D Pattern of flow of the neo-pulmonary at: early systole, peak systole, early diastole and late diastole.

The flow from the right ventricle through the very short tailored outflow to the normal pulmonary artery bifurcation showed slight acceleration of flow only at peak systole (Figure 7). During diastole there was a reversal of flow only during the late diastolic phase. This did not interfere with normal pattern of filling of the inflow of the right ventricle (Figure 7).

**Figure 8.**
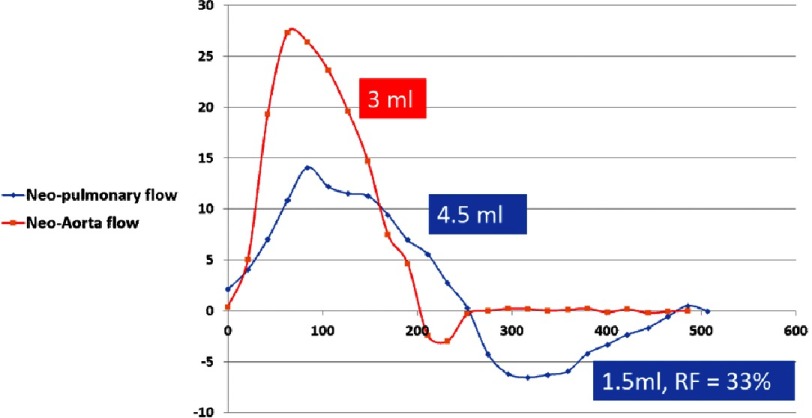
Flow profile of the neo-aorta and neo-pulmonary arteries.

The flow profile in the neo-aorta and pulmonary artery during systole shows fast flow in the neo-aorta, approximating normal aortic flow. In the pulmonary artery the flow showed slower rise and fall with reversal of flow during late diastole which amounted to approximately 1/3 of the cardiac output (Figure 8). This, however did not result in any residual volume in the right ventricle with a very small end-systolic volume (Figure 1b).

### Neo-pulmonary artery bifurcation

**Figure 9.**
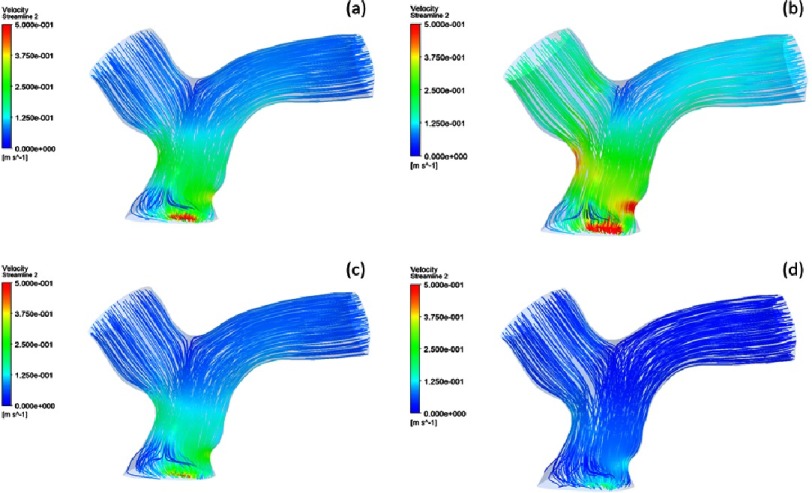
Velocity streamlines produced by CFD simulation for the neo-pulmonary artery at: early systole (a), mid-systole (b), late systole (c), and mid-diastole (d).

This was quantified by computational fluid dynamics (CFD) which showed smooth streamlines from the main pulmonary artery to both branches during systole and diastole (Figure 9).

### Conclusion

This study has shown, for the first time, the specific structural features and pattern of flow within the neo-aortic and pulmonary vessels following an innovative repair of truncus arteriosus in a patient shortly after operation. These short-term results are extremely promising and await confirmation in a larger number of patients followed up for longer periods of time.
